# Nanopower Integrated Gaussian Mixture Model Classifier for Epileptic Seizure Prediction

**DOI:** 10.3390/bioengineering9040160

**Published:** 2022-04-05

**Authors:** Vassilis Alimisis, Georgios Gennis, Konstantinos Touloupas, Christos Dimas, Nikolaos Uzunoglu, Paul P. Sotiriadis

**Affiliations:** Department of Electrical and Computer Engineering, National Technical University of Athens, 15780 Athens, Greece; giorgosyennis@gmail.com (G.G.); kostastoulou@gmail.com (K.T.); chdim@central.ntua.gr (C.D.); nikolaos.uzunoglu@gmail.com (N.U.); pps@ieee.org (P.P.S.)

**Keywords:** analog VLSI implementation, analog wake-up, epileptic seizure, Gaussian mixture model, low-power design, seizure prediction

## Abstract

This paper presents a new analog front-end classification system that serves as a wake-up engine for digital back-ends, targeting embedded devices for epileptic seizure prediction. Predicting epileptic seizures is of major importance for the patient’s quality of life as they can lead to paralyzation or even prove fatal. Existing solutions rely on power hungry embedded digital inference engines that typically consume several μW or even mW. To increase the embedded device’s autonomy, a new approach is presented combining an analog feature extractor with an analog Gaussian mixture model-based binary classifier. The proposed classification system provides an initial, power-efficient prediction with high sensitivity to switch on the digital engine for the accurate evaluation. The classifier’s circuit is chip-area efficient, operating with minimal power consumption (180 nW) at low supply voltage (0.6 V), allowing long-term continuous operation. Based on a real-world dataset, the proposed system achieves 100% sensitivity to guarantee that all seizures are predicted and good specificity (69%), resulting in significant power reduction of the digital engine and therefore the total system. The proposed classifier was designed and simulated in a TSMC 90 nm CMOS process, using the Cadence IC suite.

## 1. Introduction

The continuing progress in integrated circuit (IC) technologies has resulted in complex and power-efficient systems that address the challenges of various Internet of Things (IoT) and machine learning (ML) applications [1,2]. A particular example is wearable systems that monitor the user’s health condition, such as electroencephalogram (EEG) monitors [3]. In this case, the subject’s brain activity is monitored through the use of electrodes attached to the scalp in order to track, classify, and diagnose epileptic seizures. By continuously monitoring EEG signals throughout the everyday life of the subject, accurate conclusions about their condition can be drawn and intractable epileptic seizures [4,5], which are not amenable to medication, can be forecasted [6,7].

Wearable devices that track the EEG signals can be employed in an everyday fashion. However, the need to operate unobstructed on a Lithium battery or using energy harvesters [8] poses constraints on the acquisition procedure; all-digital signal processing and ML-powered inference can be power-hungry and limit the system’s autonomy. A trend to alleviate this limitation is the employment of cascaded classifiers, where the first ones consume relatively low power and are always on, activating more complex units only when needed [9]. Although weight quantization and pruning [10] have notably reduced the power dissipation per inference for digital ML models [11], the digital processing blocks that are provided with the models’ input features consume considerable power [12,13]. This renders the aforementioned cascaded classification scheme sub-optimal. To address this, recent work has proposed moving the feature extraction procedure in the analog part of the processing chain [12,13,14,15]. Front-end signal processing blocks, like switched capacitor filter banks, operate on the signals prior to the analog-to-digital converter (ADC) to lower the overall system’s power. The digitized features are then input to the ML model in the digital back-end.

**Figure 1:**
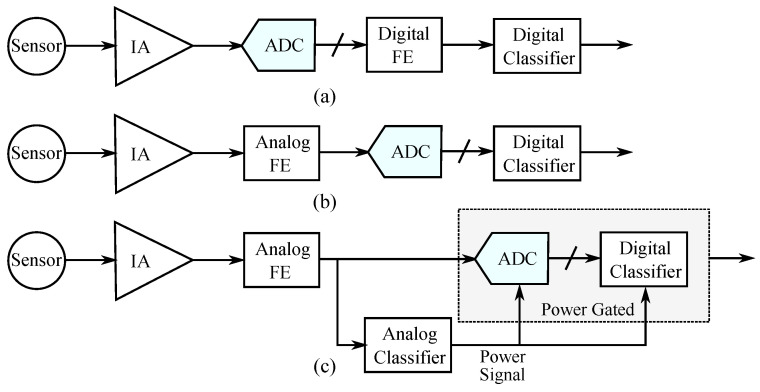
Architecture comparison. (**a**) All-digital inference. (**b**) Analog feature extraction block with a digital classifier. (**c**) Proposed concept architecture, where the digital back-end is turned on and off based on the low power analog classifier’s output.

To increase the autonomy of wearable EEG monitoring devices, overall power consumption must be decreased below the μW range. Because the energy performance of typical ML models in digital circuitry is in the μW [12,16], an alternative approach seems to be preferable. To this end, in this work we propose an ultra-low power classification system that takes advantage of analog features and uses an analog classifier as a switching device for the power-hungry digital back-end [17]. The proposed architecture, along with the mainstream approaches discussed previously, are illustrated conceptually in Figure 1. The classifier is a Gaussian mixture model (GMM), and its analog implementation consumes 180 nW of power when operating on a 0.6 V supply voltage, in the sub-threshold region. Its predictions are used to switch on and off a subsequent stage of a digital classifier, which provides high accuracy for the whole processing chain. For evaluation, the proposed classifier is designed and verified using a real-world intractable epileptic seizure dataset [4,5].

The remainder of this paper is organized as follows. The background regarding epiliptic seizure prediction and analog classifiers is provided in Section 2. Section 3 explains the mathematical foundations of GMMs. The proposed architecture and its building blocks are discussed in Section 4. Section 5 presents the experimental results of the proposed approach on a real-world EEG dataset. A comparison study and discussion are provided in Section 6. Concluding remarks are given in Section 7.

## 2. Motivation and Background

In this section we provide the necessary background on epiliptic seizure prediction and a summary of existing approaches. To introduce the reader to the state-of-the-art in analog implementations of ML systems, a summary of existing analog classifiers is also given.

### 2.1. Epileptic Seizure Prediction

An epileptic seizure is a sudden excessive neural activity or electrical disturbance in the brain [18,19].

An individual suffering from epilepsy demonstrates symptoms that vary from unnoticeable to paralyzing or even lethal. In practice, patients’ quality of life is severely affected by the unpredictability and the frequency of the seizures. A remedy to this can be prediction and warning about upcoming epileptic episodes. An accurate prediction of an upcoming seizure could allow them to prepare accordingly and avoid potentially dangerous activities, like, for instance, driving. Epileptic seizure prediction stems from examining the patients’ health using bio-signal acquisition methods.

There are four different states regarding epileptic seizures; (a) pre-ictal, (b) ictal, (c) post-ictal, and (d) inter-ictal [18,19]. States (a)–(c) refer to the periods shortly before, during, and shortly after a seizure, respectively, whereas (d) refers to the period between two seizures, when the patient is considered to be in a normal state. Based on the analysis presented in [20], the duration of the pre-ictal and post-ictal periods varies from 30 min to 2 h. An accurate and real-time identification of the pre-ictal state is crucial, as it is equivalent to predicting an upcoming seizure.

In the literature there are numerous epileptic seizure prediction systems, which follow different approaches to identify the pre-ictal periods. Although the use of EEG signals is the most common approach [20,21,22,23,24,25,26,27,28], electrocardiograph (ECG) [29,30], electromyograph (EMG) [30], heart rate [30,31], and vibration [31,32] signals have also been used. These systems achieve high accuracy (>80%) on predicting the epileptic seizures. Some of these implementations are edge computing (on-sensor computing) wearable devices [15,21,22,23], whereas others, to reduce the local power consumption, combine simple data acquisition devices with remote computing [20,24,25,26,27,28,29,30,31,32] (smartphone or cloud computing). In either case, the low power efficiency of these devices limits their capabilities and usability.

However, there exist multiple architectures that employ analog design methodologies to address epileptic seizure prediction through monitoring EEG signals. The work in [13,15] employs analog feature extraction to greatly minimize the system’s power consumption. A different approach includes employing analog pre-processing circuits directly on the acquisition device in remote computing applications [33,34,35]. In particular, the analog circuit reduces the overall power consumption of the communication device by reducing the data that need to be transferred to the remote server for prediction. A brief summary of seizure prediction systems in terms of employed algorithms, operating device, and power consumption for all the aforementioned implementations is provided in Table 1.

### 2.2. Analog Classifiers

Analog integrated circuits (ICs), powered by their capability to operate in the sub-threshold domain [36], are gaining popularity as a means to reduce power consumption in comparison to their digital counterparts. Applications that employ real-time ML techniques are typically power hungry and could greatly benefit from analog circuitry. Nonetheless, analog circuits struggle with high dimensional classification problems as they typically require multiple cascaded multipliers. In practice, analog multipliers are usually unreliable and their operating voltage range is limited. Two main approaches regarding this issue are to either tailor multipliers for specific applications [37,38] or utilize architectures and/or circuits that avoid multipliers [39,40,41,42,43]. Following the former approach, translinear-based, current-mode multipliers [44] are the most popular choice. Regarding the latter, Gaussian function circuits are a commonly used solution [45].

Translinear-based Gaussian function circuits [45] that consist of squaring and exponentiator circuits are utilized in [39,40]. In this case, by leveraging the properties of the exponential function, the multiplication is replaced by the summation of the exponents, which is a trivial task. Alternatively, the work proposed in [41,42,43] uses more compact building blocks, e.g., bump circuits [41], that implement multivariate Gaussian functions without the use of multipliers. A performance summary of the aforementioned work is presented in Table 2.

By examining Table 2, the implementation with the lowest power consumption is [41] (365 nW). This is due to the combination of a compact and simple ML model with ultra low-power building blocks that operate in the sub-threshold domain with a low supply voltage. Based on our previous work in [41], here we build a low power analog classifier and improve upon the accuracy by employing a GMM model instead of a simple Gaussian one.

## 3. Gaussian Mixture Model

In this section, the mathematical foundations of GMMs, which comprise the core of the proposed classifier, are given. In addition, the use of the GMMs within the scope of classification is also described.

Consider an *N*-dimensional random variable $X=\left[x_{1},\ldots ,x_{n}\right]$ and its probability density function (PDF) *p* with X∼p. The GMM is a probabilistic model that consists of a weighted sum of Gaussian distributions and can be used to approximate unknown PDFs from data [46]. In the case of X, the GMM’s Gaussian distributions, also noted as components, are also *N*-dimensional. GMMs belong to the general class of mixture models (MMs) and are widely used in the literature, as they combine both the approximation capabilities of MMs and the properties of Gaussian distributions.

The approximate PDF of X, as modeled by a GMM λ, is given by

(1) $$p\left(X|\lambda \right)=\sum_{i=1}^{K}w_{i}\cdot N\left(X|M_{i},\Sigma_{i}\right).$$

Here, the component count is K≥1 and for the weights it holds that $\sum_{i=1}^{K}w_{i}=1$. Each of the components is an *N*-dimensional Gaussian distribution with a (N×1) mean vector $M_{i}$ and a (N×N) covariance matrix $\Sigma_{i}$, for i=1,…,K.

In the special case of diagonal covariance matrices, each Gaussian distribution is given by

(2) $$N\left(X|M_{i},\Sigma_{i}\right)=\prod_{n=1}^{N}N\left(x_{n}|\mu_{n}^{i},{\left(\sigma_{n}^{i}\right)}^{2}\right),$$

where superscript ‘*i*’ denotes the Gaussian component and subscript ‘*n*’ the dimension, i.e., $\mu_{n}^{i}$ is the *n*th component of vector $M_{i}$ and $\sigma_{n}^{i}$ is the *n*th component of the diagonal of matrix $\Sigma_{i}$. Hence, each component is derived by the product of *N* univariate Gaussian distributions given by

(3) $$N\left(x_{n}|\mu_{n},{\left(\sigma_{n}\right)}^{2}\right)=\frac{1}{\sqrt{\left(2\pi \right)\cdot {\left(\sigma_{n}\right)}^{2}}}\;e^{-\frac{1}{2}\cdot \frac{{\left(x_{n}-\mu_{n}\right)}^{2}}{{\left(\sigma_{n}\right)}^{2}}}.$$

GMMs are adapted to data by using the expectation-maximization (EM) algorithm [46]. Although their unsupervised nature renders them suitable for clustering problems, they can also be used within the scope of supervised classification models. Considering a dataset D with *N*-dimensional input vectors and *C* classes, one can fit *C* separate GMMs ${\left[\lambda_{i}\right]}_{i=1}^{C}$ to each subset of D associated with each class. Therefore, the PDF of the input vectors that belong to each class is approximated by a GMM.

Using the above setting, one can infer the class *y* of a new input vector X of an unknown class as the one whose approximate PDF provides the highest likelihood, i.e.,

(4) $$\begin{array}{c} \begin{array}{cc} y & =\underset{c\in [1,C]}{\mathrm{argmax}}\left\{p(X|\lambda_{c})\right\}\\ \; & =\underset{c\in [1,C]}{\mathrm{argmax}}\left\{\sum_{i=1}^{K}w_{i}^{c}\cdot N\left(X|M_{i}^{c},\Sigma_{i}^{c}\right)\right\}. \end{array} \end{array}$$

In this case, superscript ‘*c*’ denotes the class. It is important to note that in Equation (4) all GMMs share the same number of components *K*. In the supervised setting, *K* is denoted as clusters, and this is the naming this paper follows for the rest of the sections. The number of clusters is a hyperparameter of the overall classifier and it is chosen based on the complexity of the data.

## 4. Proposed Architecture

In this section, the architecture of the proposed analog classifier and the operation of its building blocks are analyzed. To reduce the overall power consumption, in the following building blocks, all transistors operate in the sub-threshold region, and the power supply rails are set to $V_{DD}=-V_{SS}=0.3V$ for the entire classifier.

Based on Section 3, a GMM-based classifier requires two basic building blocks: one that generates a Gaussian PDF, as in (1), and another that implements the argmax operator, as in (4). In the case of analog hardware, bump circuits have been proposed for the hardware implementation of a univariate Gaussian PDF [47]. Recently, a modified version of the bump circuit was proposed to generate multivariate PDFs as well [48]. Concerning the analog implementation of the argmax operator, winner-take-all (WTA) circuits have been employed in the literature [49]. In this work, we modify a typical bump circuit and use it in the proposed classifier in order to increase its accuracy.

**Figure 2:**
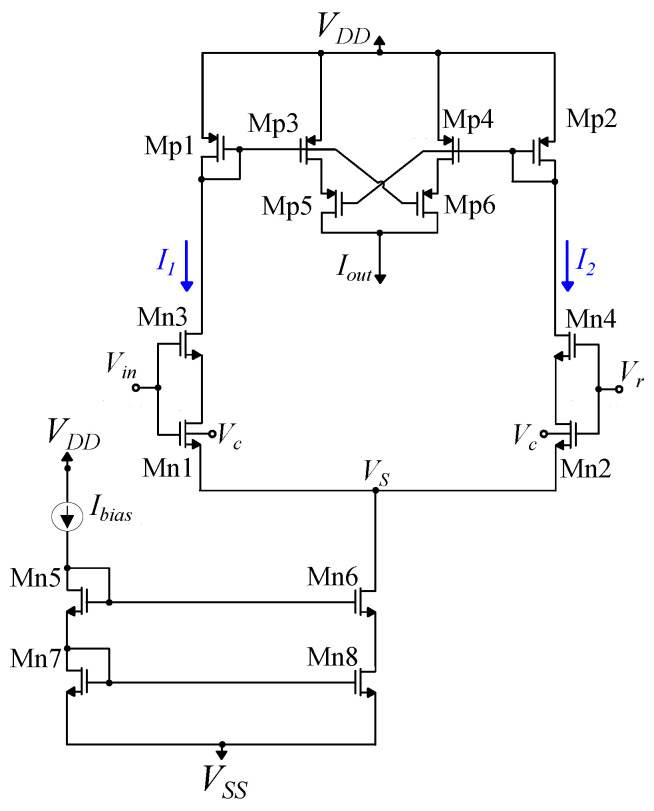
The proposed analog architecture implementing a Gaussian function (bump circuit). $V_{in}$, $V_{r}$, $V_{c}$, and $I_{bias}$ are the input voltage, the voltages controlling the mean value, and the variance and the bias current controlling the height of the Gaussian curve, respectively.

**Figure 3:**
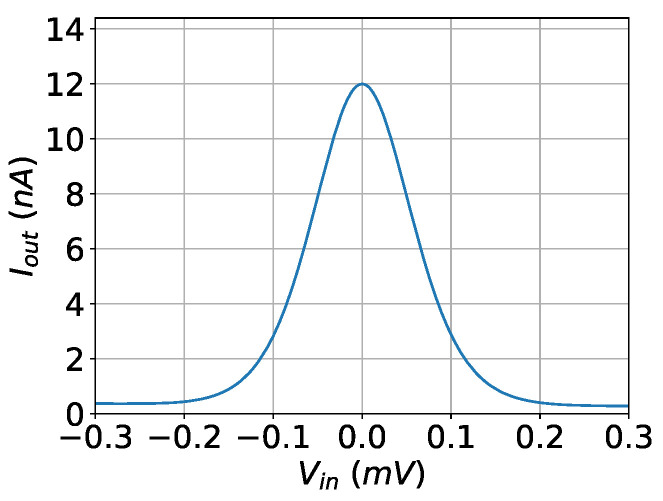
The output of the bump circuit for $I_{bias}=12$ nA, $V_{r}=0$ V, and $V_{c}=0$ V.

The modified bump circuit is a combination of two sub-circuits: a symmetric current correlator [48] and a differential block [50]. The aim of this modification is to increase the quality of the Gaussian curve and reduce the distortion in the case of the multivariate bump circuits. In particular, the symmetric current correlator improves the symmetry of the Gaussian curve around the mean value [48]. The simple differential block offers good control of the Gaussian curve’s parameters with a minimal area [50]. The cascode mirrors are used instead of the standard ones, to offer robust mirroring even for small bias currents. This is necessary for multivariate bump circuits. This bump circuit, shown in Figure 2, provides a more accurate Gaussian curve, shown in Figure 3, than either of [48,50]. Transistors’ dimensions are summarized in Table 3. The mean value, the variance, and the height of the Gaussian curve are controlled via the voltage parameters $V_{r}$ and $V_{c}$ and the bias current $I_{bias}$, respectively [48,50].

**Figure 4:**
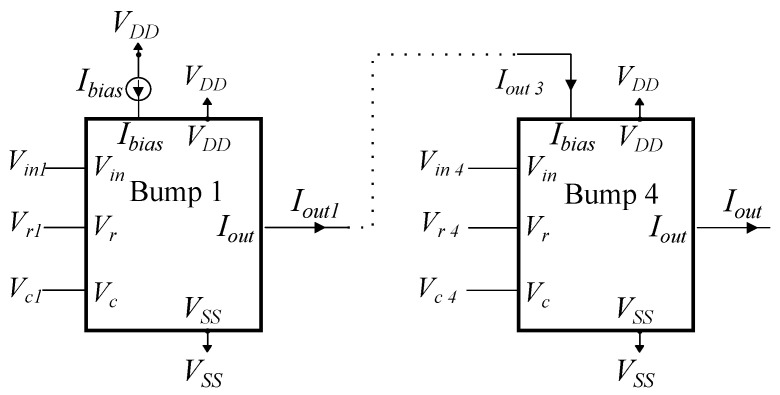
A 4D Bump circuit implementation composed of four sequentially connected univariate bump circuits.

The multivariate Gaussian PDF is realized by multiplying two or more bump circuits, as described in Equation (2). Consider a sequence of two bump circuits. Biasing the second one with the output current of the first one results in an overall output current that is equivalent to the multiplication of their respective Gaussian curves [48]. An implementation of a 4D Gaussian PDF (four cascaded bump circuits) is shown in Figure 4. Only the first bump is biased with a preset current ($I_{bias}$), representing the weight $w_{i}$ of the corresponding cluster *i*. The topology in Figure 4 constitutes a cluster of the proposed GMM-based classifier.

**Figure 5:**
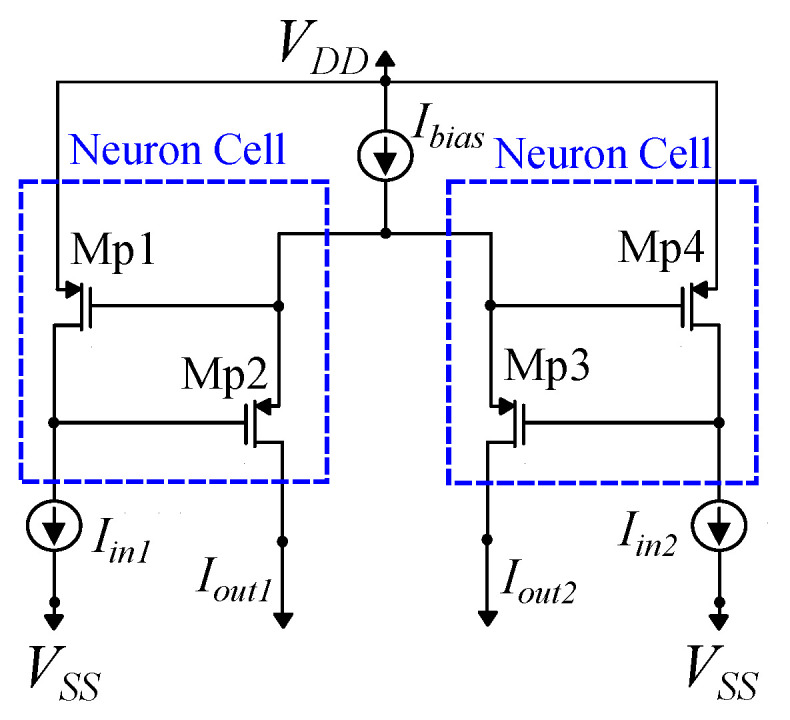
The Lazzaro WTA circuit composed of two PMOS-based neuron circuits.

The second block of the proposed architecture is a Lazzaro WTA circuit [49]. Its flexibility and simplicity make it the most popular choice for the implementation of the argmax operator. This WTA circuit is composed of sub-blocks denoted as neuron cells. For a *C* class classification problem the number of neuron cells must be also *C*, each one responsible for a single class. In particular, each neuron cell receives the likelihood from a specific GMM and outputs a current in binary format; if the GMM corresponds to the class with the highest likelihood, this current is logical one (which is close to the WTA’s bias current), otherwise it is logical zero (less than 100 pA). For demonstration purposes, a transistor level implementation of a WTA circuit with two neurons is shown in Figure 5. All transistors’ dimensions are equal to W/L=0.4μm/1.6 μm.

**Figure 6:**
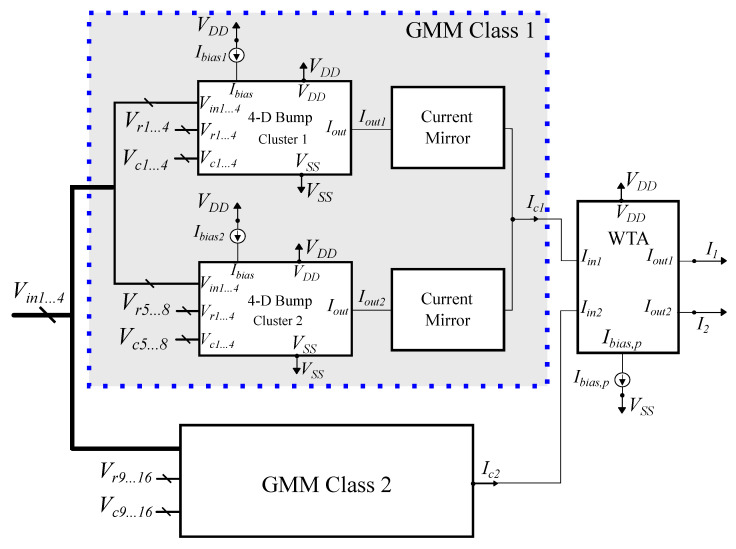
Analog GMM-based classifier with two classes, two clusters per class, and 4D inputs (four input features). (**left**) GMM class with two clusters; (**right**) WTA circuit. GMM class 2 follows the same architecture as the depicted GMM class 1.

Utilizing the aforementioned building blocks and based on Equation (4), the proposed GMM-based classifier with two classes, two clusters per class, and 4D inputs is shown in Figure 6. Each GMM class is comprised of two 4D bump circuits, which correspond to the two clusters, and two current mirrors that are used to add the output currents of each cluster. The overall output current ${\left[I_{ci}\right]}_{i=1}^{2}$ of each class is analogous to the class’ likelihood. The WTA circuit compares these probabilities and the predicted class is determined via the currents ${\left[I_{i}\right]}_{i=1}^{2}$.

It should be noted that it is impractical to provide the classifier’s 34 controlling parameters (${\left[V_{rj}\right]}_{i=j}^{16}$, ${\left[V_{cj}\right]}_{j=1}^{16}$ and ${\left[I_{biasi}\right]}_{i=1}^{2}$) externally. Therefore, an alternative option that involves integrating analog memories adjacent to the classifier is preferable. In particular, as typically the classifier will be trained only once prior to its deployment, non-volatile analog memories are a promising choice [51,52]. However, for a general purpose classifier that may require altering this configuration multiple times, dynamic memories can be a more opportune solution [53,54].

## 5. Epileptic Seizure Prediction Application

**Figure 7:**
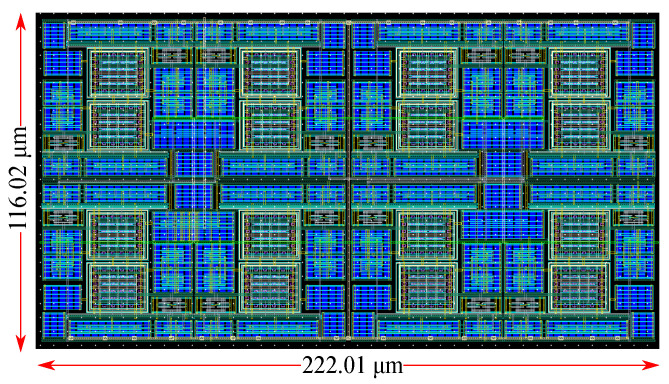
Layout of the proposed classifier circuit.

In this section, the proposed classifier is tested on a real-world epilepsy seizure prediction problem [4,5] to confirm its proper operation. The classifier has been designed using the Cadence IC suite in a TSMC 90 nm CMOS process. All simulation results are conducted on the layout (post-layout simulations), which is shown in Figure 7.

The data are acquired from the CHB-MIT Scalp EEG database [4,5] and contain EEG signals from children with intractable epilepsy. The ictal periods are labeled by expert physicians. Here, pre-ictal and post-ictal periods are considered to span an hour before and an hour after the seizure, respectively. The data samples that do not belong in ictal, pre-ictal, or post-ictal periods are labeled as inter-ictal.

There are four features for the classification: the signal’s peak-to-peak voltage and energy percentages in the alpha and the first and second half of the gamma frequency bands [55]. These features can be efficiently derived from the raw EEG signals using analog feature extraction techniques [13,56]. The system’s necessary parameters are derived by software-based training, prior to the circuit’s deployment.

The aim of the classifier is to successfully distinguish the pre-ictal from the inter-ictal periods. In order to operate as a minimal power front-end wake-up circuit, it must predict all possible seizures and maintain a low number of false positive alarms. The first requirement is equivalent to having high classification sensitivity [57], which is measured by:

(5) $$\mathrm{sensitivity}=\frac{\mathrm{Predicted}\;\mathrm{Seizures}}{\mathrm{Predicted}\;\mathrm{Seizures}+\mathrm{Missed}\;\mathrm{Seizures}}.$$

Achieving a high sensitivity score is crucial for the patient’s health, as it ensures that all upcoming seizures will be predicted. However, the second requirement is equivalent to minimizing the rate with which the high power consumption digital back-end is turned on. This leads to a significant power consumption reduction for the whole system, shown in Figure 1c. An appropriate measure to quantify this reduction is the specificity [57] of the analog classifier, given by:

(6) $$\mathrm{specificity}=\frac{\mathrm{True}\;\mathrm{Negative}}{\mathrm{True}\;\mathrm{Negative}+\mathrm{False}\;\mathrm{Positive}}.$$

In practice, this metric is the ratio of the time that the digital back-end is idle to the duration of all the inter-ictal periods (no risk for seizure).

**Figure 8:**
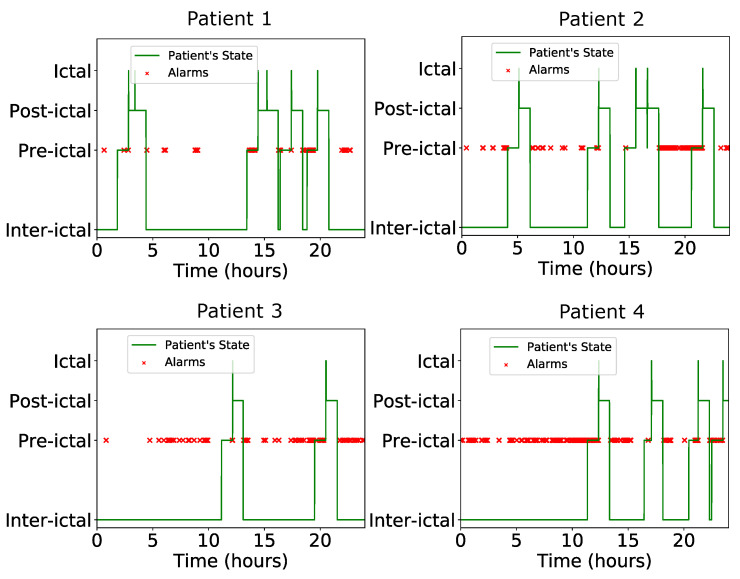
The alarms triggered by the classifier for four patients in a 24-h period. The ideal behavior is the raising of at least one alarm in each pre-ictal period, without raising alarms during the inter-ictal ones. The ictal and post-ictal regions are irrelevant for the classifier.

**Figure 9:**
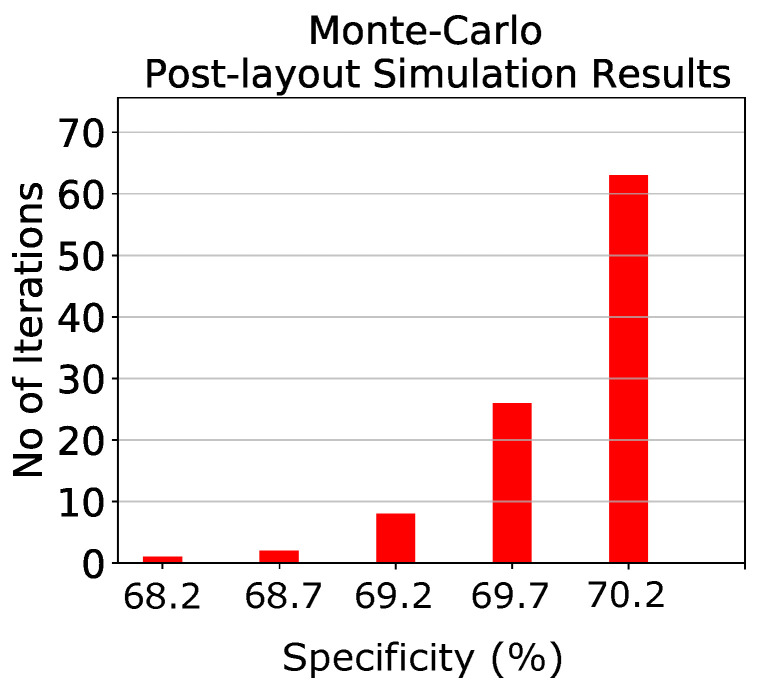
Post-layout Monte-Carlo sensitivity analysis simulation results on the specificity of the classifier for one of the previous 20 iterations.

To test the proposed classifier both in terms of classification specificity and circuit’s behavior in PVT variations, two separate tests are conducted. The first one is a comparison between the proposed implementation and a software-based one. In particular, 20 separate software-based training iterations are conducted to account for random effects. The resulting specificity scores are summarized in Table 4. The proposed architecture’s mean specificity is only 2% lower than that of a software-based implementation. For demonstration purposes, the state of four patients along with the predictions of the analog classifier are presented in Figure 8. The classifier successfully predicts all 17 seizures (100% sensitivity) of the test set. The second test is a Monte-Carlo analysis for N=100 points, for one of the previous 20 candidates. The Monte-Carlo analysis histogram is shown in Figure 9. Its mean value is $\mu_{M}$ = 69.93% with a standard deviation of $\sigma_{M}$ = 0.41%. This confirms the proper performance and operation of the proposed architecture.

## 6. Discussion and Comparison

A comparison between this work and other studies that employ analog design methodologies to address epileptic seizure prediction through monitoring EEG signals is provided in Table 5. Here, it is seen that this work achieves very low power consumption per channel (180 nW per channel), outperforming all the implementations except that from [13], which achieves 96 nW per channel. Nonetheless, as the proposed implementation requires only a single channel, its total power consumption is significantly smaller. In particular, the proposed architecture consumes power in the range of nW, which is not the case for the rest of the implementations in Table 5. This power dissipation is achieved using a supply voltage of only 0.6 V, which is also the lowest one in Table 5. The specificity of the proposed classifier is 69%, which, along with [13] (86%) and [15] (84.4%), constitutes the three highest specificity scores.

Another important metric for measuring efficiency in analog computing, which is invariant to the application and is therefore a relatively fair metric for comparing architectures designed for different applications, is the energy consumed per operation. The proposed classifier consumes 180 nW and can achieve a computational speed of 166 K classifications per second, which results in 1.1 pJ per classification. Each classification, for a GMM-based classifier composed of two classes, two clusters per class, and 4D inputs, requires 131 operations. This results in the classifier’s consumption being 8.2 fJ per operation. Unfortunately, these metrics are not provided in the literature for comparison purposes.

As shown in Table 5, most epileptic seizure prediction systems employ multiple channels, i.e., electrodes, in order to increase their accuracy. Nonetheless, acquisition devices with multiple electrodes are usually uncomfortable for the patient and impractical for constant monitoring. To this end, this work focuses on extracting data from a single electrode. By doing so, the resulting device is less bulky and more convenient to use. In addition, to further increase the device’s portability, the classifier is proposed to operate in an embedded device. In this way, the patient can be monitored constantly with no requirements for wireless communication with other devices as proposed in [33,34,35].

In real-world scenarios, EEG signal acquisition is affected by uncontrolled parameters and environmental factors. In the case of a single electrode in particular, motion artifacts, electrode misplacement, and external electromagnetic interference can drastically reduce the quality of the signal and potentially lead to diagnostic errors. Having multiple electrodes for signal acquisition may seem more robust, as the contaminated recordings could be only a fraction of the total inputs to the prediction system, but this comes at the cost of the system’s portability and with no theoretical guarantee. Efforts to determine the goodness of the acquired signals have been proposed in the literature via employing ML classification techniques [58]. We argue that this quality assessment can implicitly take place within the GMM classifier of our system provided that: (a) real-world, noise-contaminated EEG signals are used for training and, (b) the classifier is expanded to provide confidence bounds about its predictions. By doing so, additional systems for quality assessment, as in [58], can be avoided and thereby the area and power consumption of the device is unchanged.

Another important design consideration is the trade-off between the wake-up circuit’s power consumption and its specificity. As the specificity of the wake-up circuit increases, the overall power consumption of the digital circuit, which is typically greater than the analog one’s, decreases. However, to achieve high specificity values, it is essential to increase the complexity of the analog circuit. In particular, to improve the classifier’s performance, improved-performance acquisition devices, more analog feature extraction circuits, and larger analog memories storing the classifier’s parameters are required. All the aforementioned modifications result in increased power consumption. In practice, increasing the power consumption of the analog front-end must be done cautiously; a classification system with a power greedy analog classifier that switches on and off a digital one may consume more power than an all-digital one.

## 7. Conclusions

A fully analog processing unit was presented as an alternative to the conventional front-end architectures for inference systems targeting EEG signals. The proposed system includes an 180 nW or 8.2 fJ per operation analog integrated GMM-based classifier, which activates the high-performance digital inference back-end only when needed. Its main building blocks are Gaussian function circuits and the Lazzaro WTA circuit. The classifier was trained on a real-world seizure prediction dataset and designed in a TSMC 90 nm technology. Post-layout simulation results suggest that the proposed circuit achieves 100% sensitivity, as all 17 seizures of the test set are predicted, and 69.07% specificity.

## Figures and Tables

**Table 1:** Performance summary for epileptic seizure prediction systems.

Ref.	Model	Device	Power Related Metric
[13]	SVM	hardware	3.07 μW
[15]	SVM	hardware	66 μW
[20]	LSTM	software	N/A
[21]	SVM	hardware	$7.13\frac{\mathrm{uJ}}{\mathrm{feature}}$
[22]	SVM	hardware	$1.35\frac{\mathrm{uJ}}{\mathrm{classification}}$
[23]	Perceptron	hardware	55.89 mW/ 25 h
[24]	VAE	software	N/A
[25]	CNN	software	N/A
[26]	SVM	software	N/A
[27]	multi-model	smartphone	N/A
[28]	ANN	software	N/A
[29]	custom	(MCU) MSP430 and Cloud	N/A
[30]	custom	smartphone	N/A
[31]	custom	arduino	N/A
[32]	SDA	cloud	N/A
[33]	filters	hardware	30.4 μW
[34]	filters	hardware	3.65 mW
[35]	filters	hardware	23 μW

**Table 2:** Performance summary for analog classifiers.

Ref.	Technology	Model	Dimensions	Power Consumption	Area
[37]	0.5 μm	SVM	14	840.0 nW	9.000 mm${}^{2}$
[38]	180 nm	LSTM	16 × 16 matrix	460.3 mW	9.990 mm${}^{2}$
[39]	180 nm	SVM	64	N/A	0.125 mm${}^{2}$
[40]	180 nm	K-means	164	N/A	N/A
[41]	90 nm	Bayesian	5	365 nW	0.030 mm${}^{2}$
[42]	180 nm	SVM	2	220.0μW	0.060 mm${}^{2}$
[43]	0.5μm	RBF NN	2	N/A	2.250 mm${}^{2}$

**Table 3:** MOS transistors’ dimensions (Figure 2).

Differential Block	W/L (μm/μm)	Current Correlator	W/L (μm/μm)
$M_{n1}$,$M_{n2}$	2.0/1.0	$M_{p1}$,$M_{p2}$	0.8/1.6
$M_{n3}$,$M_{n4}$	2.0/0.1	$M_{p3}$–$M_{p6}$	0.4/1.6
$M_{n5}$–$M_{n7}$	0.4/1.6	-	-
$M_{n8}$	1.6/1.6	-	-

**Table 4:** Specificity (over 20 iterations).

Method	Best	Worst	Mean	Std.
Software	71.30%	71.08%	71.27%	0.07%
Proposed	70.65%	67.39%	69.07%	0.51%

**Table 5:** Performance summary for analog epileptic seizure prediction systems.

Ref.	Technology	Power Supply	Power Consumption per Channel	Total Power Consumption	No. of Channels	Specificity
This work	90 nm	0.6 **V**	180 nW	180 **nW**	1	69%
[13]	65 nm	N/A	96 **nW**	3.07 μW	23	86%
[15]	180 nm	1.8 V	8.25 μW	66 μW	8	84.4%
[33]	350 nm	1.25 V	950 nW	30.4 μW	32	55%
[34]	90 nm	1.25 V	1.14 μW	3.65 mW	32	48.5%
[35]	180 nm	1.8V	23 μW	23 μW	1	50%

## Data Availability

The data used in this study are openly available in CHB-MIT Scalp EEG Database at https://physionet.org/content/chbmit/1.0.0/ (accessed on 21 March 2022), reference number [4].
